# The role of CDX2 in renal tubular lesions during diabetic kidney disease

**DOI:** 10.18632/aging.202537

**Published:** 2021-02-17

**Authors:** Huiming Liu, Rui Yan, Luqun Liang, Huifang Zhang, Jiayi Xiang, Lingling Liu, Xiaohuan Zhang, Yanwen Mao, Wei Peng, Ying Xiao, Fan Zhang, Yuxia Zhou, Mingjun Shi, Yuanyuan Wang, Bing Guo

**Affiliations:** 1 Department of Pathophysiology, Guizhou Medical University, Guiyang 550025, Guizhou, China; 2 Guizhou Provincial Key Laboratory of Pathogenesis and Drug Research on Common Chronic Diseases, Guizhou Medical University, Guiyang 550025, Guizhou, China; 3 Department of Nephrology, Affiliated Hospital of Guizhou Medical University, Guiyang 550025, Guizhou, China; 4 Department of Pathology, West China Hospital, Sichuan University, Chengdu 610041, Sichuan, China

**Keywords:** CDX2, diabetic kidney disease, CFTR, Wnt/β-catenin signaling, renal tubular lesions

## Abstract

Renal tubules are vulnerable targets of various factors causing kidney injury in diabetic kidney disease (DKD), and the degree of tubular lesions is closely related to renal function. Abnormal renal tubular epithelial cells (RTECs) differentiation and depletion of cell junction proteins are important in DKD pathogenesis. Caudal-type homeobox transcription factor 2 (CDX2), represents a key nuclear transcription factor that maintains normal proliferation and differentiation of the intestinal epithelium. The present study aimed to evaluate the effects of CDX2 on RTECs differentiation and cell junction proteins in DKD. The results demonstrated that CDX2 was mainly localized in renal tubules, and downregulated in various DKD models. CDX2 upregulated E-cadherin and suppressed partial epithelial-mesenchymal transition (EMT), which can alleviate hyperglycemia-associated RTECs injury. Cystic fibrosis transmembrane conductance regulator (CFTR) was regulated by CDX2 in NRK-52E cells, and CFTR interfered with β-catenin activation by binding to Dvl2, which is an essential component of Wnt/β-catenin signaling. CFTR knockdown abolished the suppressive effects of CDX2 on Wnt/β-catenin signaling, thereby upregulating cell junction proteins and inhibiting partial EMT in RTECs. In summary, CDX2 can improve renal tubular lesions during DKD by increasing CFTR amounts to suppress the Wnt/β-catenin signaling pathway.

## INTRODUCTION

DKD, one of the main complications of diabetes, represents the most important factor leading to end-stage renal disease (ESRD) and kidney transplantation [1]. Although glomerular damage is the main pathological feature of DKD, multiple reports have shown that tubular injury is critical in DKD, and the degree of renal tubular injury is closely related to renal function [2, 3]. Loss or reduction of the cell adhesion protein E-cadherin constitutes an important initiation factor of RTECs shedding, which impairs barrier function and results in kidney inflammation and fibrosis [4]. In addition, partial EMT of RTECs represents an important pathological mechanism of tubulointerstitial fibrosis in DKD [5]. Therefore, upregulating cell junction proteins and inhibiting partial EMT are key events in preventing DKD.

CDX2, a gut nuclear transcription factor, regulates the differentiation, proliferation and maintenance of intestinal epithelial cells [6]. Studies assessing CDX2 are mainly related to digestive tract tumors, and reveal an oncogenic role for CDX2 in esophageal cancer [7], but a suppressor function in colorectal [8], gastric [9], and breast [10] cancers. Previous evidence shows CDX2 inhibits gastric cancer cell invasion and metastasis by upregulating cell junction protein E-cadherin and Claudin-2 [11, 12]. However, CDX2’s expression and function in DKD remain undefined.

Cystic fibrosis transmembrane conductance regulator (CFTR), a Cl- channel protein induced by cAMP, is broadly found in epithelial cells of various tissues [13]. Recent evidence shows that CFTR inhibits UUO-induced renal fibrosis by affecting Wnt/β-catenin signaling [14]. Meanwhile, Wnt/β-catenin signaling has a critical function in DKD development [15]. Indeed, β-catenin is activated and undergoes nuclear translocation, which promotes the transcription of Snail that can directly mediate partial EMT and loss of E-cadherin [16], thereby leading to tubulointerstitial fibrosis [17, 18]. In intestinal epithelial cells, CDX2 interacts with the intronic elements of CFTR and enhances its transcriptional activity, thereby elevating the protein expression of CFTR [19, 20]. Therefore, this work aimed to assess CDX2 expression in the kidney and explore its possible mechanism in DKD.

## RESULTS

### CDX2 is downregulated in DKD

**Figure 1:**
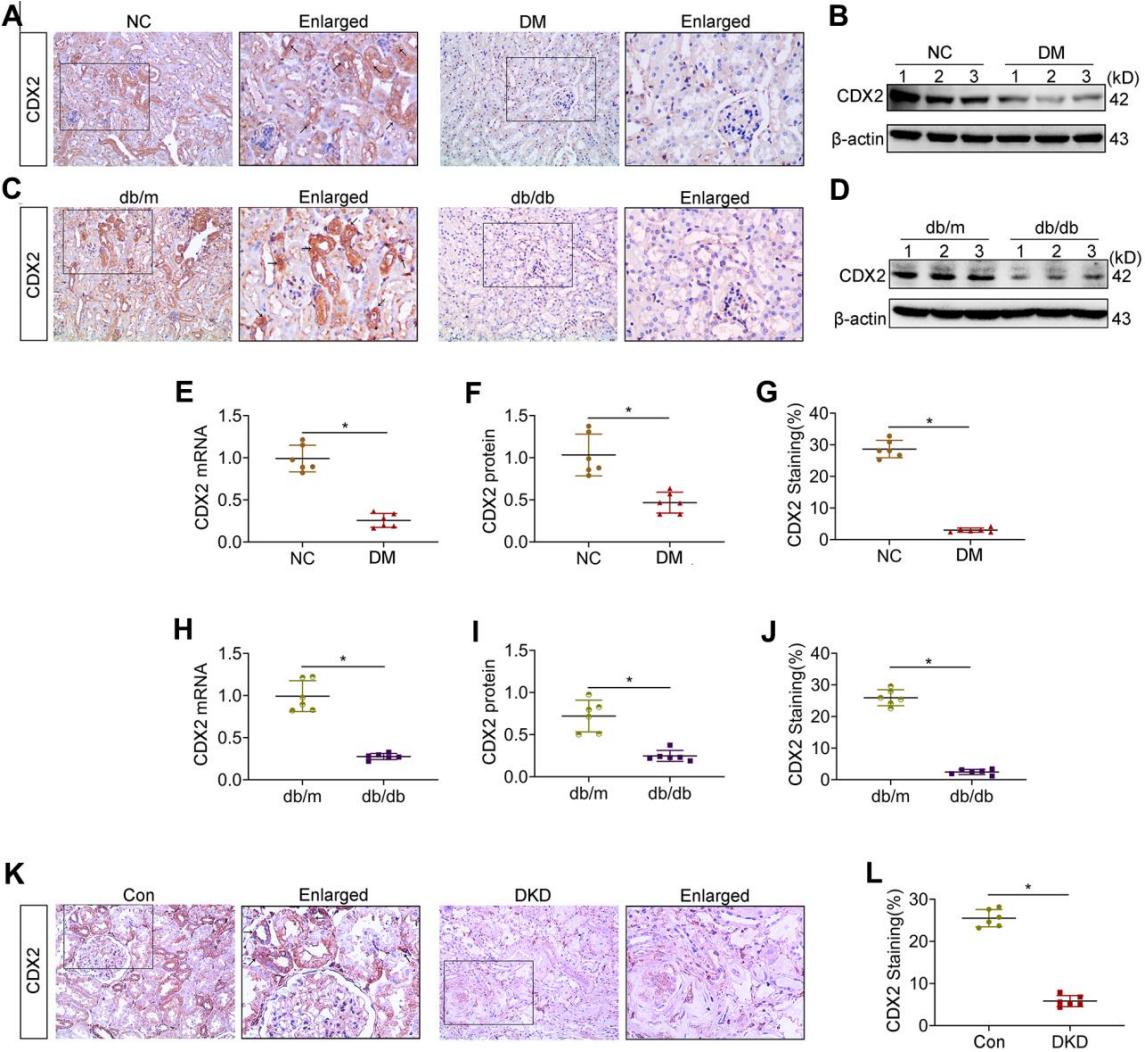
**Kidney CDX2 is downregulated in DKD.** T1D mice (DM group) and controls were submitted to euthanasia at 16 weeks of age (6 weeks after the establishment of the T1D mouse model), and T2D mice (db/db group) and controls were submitted to euthanasia at 18 weeks of age. (**A**, **B**) Immunohistochemical staining (**A**) and immunoblot (**B**) for CDX2 detection in T1D mice and controls. (**C**, **D**) Immunohistochemical staining (**C**) and immunoblot (**D**) for CDX2 detection in T2D mice and controls. (**E**–**G**) Immunohistochemical-positive staining density of CDX2 was analyzed in each group from 6 random fields (200×). Quantitation of mRNA amounts (**E**), Western blot bands (**F**) and immunohistochemical signals (**G**) of CDX2 in T1D mice kidney tissues and controls. (**H**–**J**) Quantitation of mRNA amounts (**H**), Western blot bands (**I**) and immunohistochemical signals (**J**) of CDX2 in T2D mice kidney tissues and controls. (**K**, **L**) Immunohistochemical staining (**K**) and quantitative analysis (**L**) of CDX2 in DKD patients kidney tissues and controls. CDX2 is expressed in the cytoplasm and nucleus of renal tubular epithelial cells in the renal cortex (black arrow) (magnification, ×200); enlarged box area (magnification,×400). All data are mean±SD from three independent experiments. n=6; ^*^P<0.05.

In order to assess renal CDX2 expression in DKD, kidney CDX2 amounts were determined. Representative photomicrographs demonstrated that CDX2 was highly expressed in non-diseased mice and human kidney tissues, and mainly located in the cytosol and nucleus of RTECs (arrow), but decreased in kidney samples from T1D and T2D mice as well as DKD patients (Figure 1A, 1C–1K). Similarly, CDX2 protein amounts were significantly decreased in kidney specimens from T1D and T2D animals compared with the corresponding controls (Figure 1B–1D). Additionally, renal CDX2 mRNA amounts were significantly reduced in T1D and T2D mice compared with controls (Figure 1F–1H). kidney CDX2 is downregulated in IgA nephropathy and in UUO (unilateral ureteral obstruction) (Supplementary Figure 1). These results suggested that renal CDX2 was reduced in DKD and other chronic kidney diseases.

### CDX2 is involved in the development of DKD

**Figure 2:**
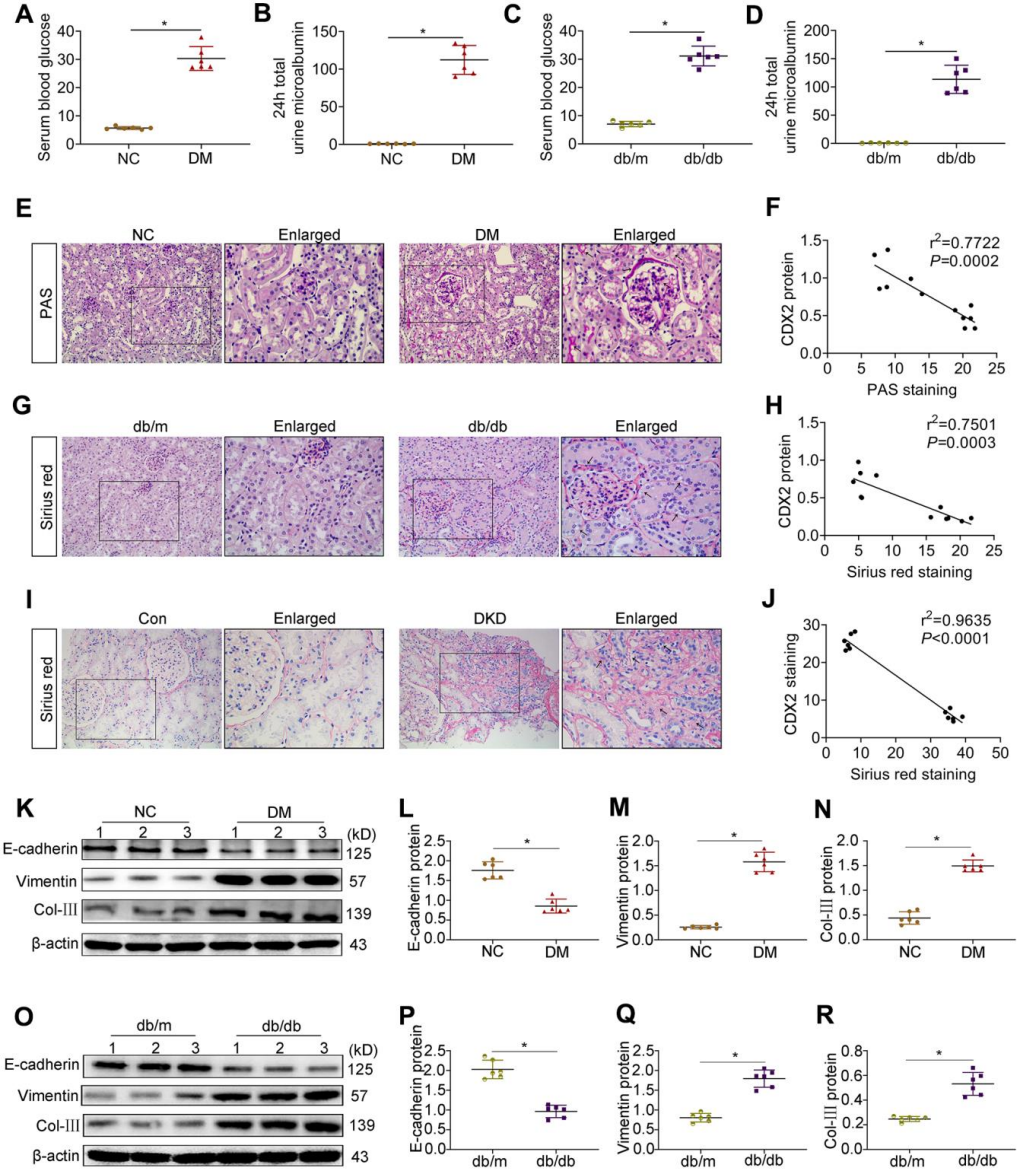
**CDX2 is negatively related to the development of DKD.** (**A**, **B**) Blood glucose (**A**) and 24h total urine microalbumin (**B**) were significantly increased in T1D mice than controls. (**C**, **D**) Blood glucose (**C**) and 24h total urine microalbumin (**D**) were significantly increased in T2D mice than controls. Positive staining density of PAS and Sirius Red was analyzed in each group from 6 random fields (200×). (**E**, **F**) PAS staining (**E**), correlation of CDX2 staining with PAS staining in the kidneys of T1D mice and controls (**F**, *r* = 0.7722; *P* = 0.0002). (**G**, **H**) Sirius Red staining (**G**), correlation of CDX2 staining with Sirius Red staining in kidneys of T2D mice and controls (**H**, *r* = 0.7501; *P* = 0.0003). (**I**, **J**) Sirius Red staining (**I**), correlation of CDX2 staining and Sirius Red staining in the kidneys of DKD patients and controls (**J**, *r* = 0.9635; *P* = 0.0001). (**K**–**N**) Immunoblot bands of E-cadherin, Vimentin, and Col-III in T1D mice and controls (**K**), quantitative data are presented (**L**–**N**). (**O**–**R**) Immunoblot bands of E-cadherin, Vimentin, and Col-III in T2D mice and controls (**O**); quantitative data are presented (**P**–**R**). Data are mean±SD from three independent assays. *n*=6; ^*^P<0.05 versus NC group or db/m group or Con group.

Both T1D and T2D mice showed the important feature of diabetes (hyperglycemia) as well as the characteristic manifestation of DKD (microalbuminuria) (Figure 1A–1D). PAS staining and Sirius Red staining showed mesangial matrix expansion, glomerular hypertrophy, vacuolar formation of renal tubules, and extracellular matrix accumulation (Figure 2E–2G). The renal photomicrographs of DKD patients revealed glomerulosclerosis and tubulointerstitial fibrosis (Figure 2I). Compared with control values, E-cadherin protein amounts were decreased while Vimentin and Col-III levels were increased in the kidneys of T1D and T2D mice (Figure 2K–2O). Correlation analysis showed that CDX2 protein amounts had a negative correlation with the degree of kidney injury (Figure 2F, 2H, 2J).

### CDX2 relieves hyperglycemia-associated renal tubular injury

**Figure 3:**
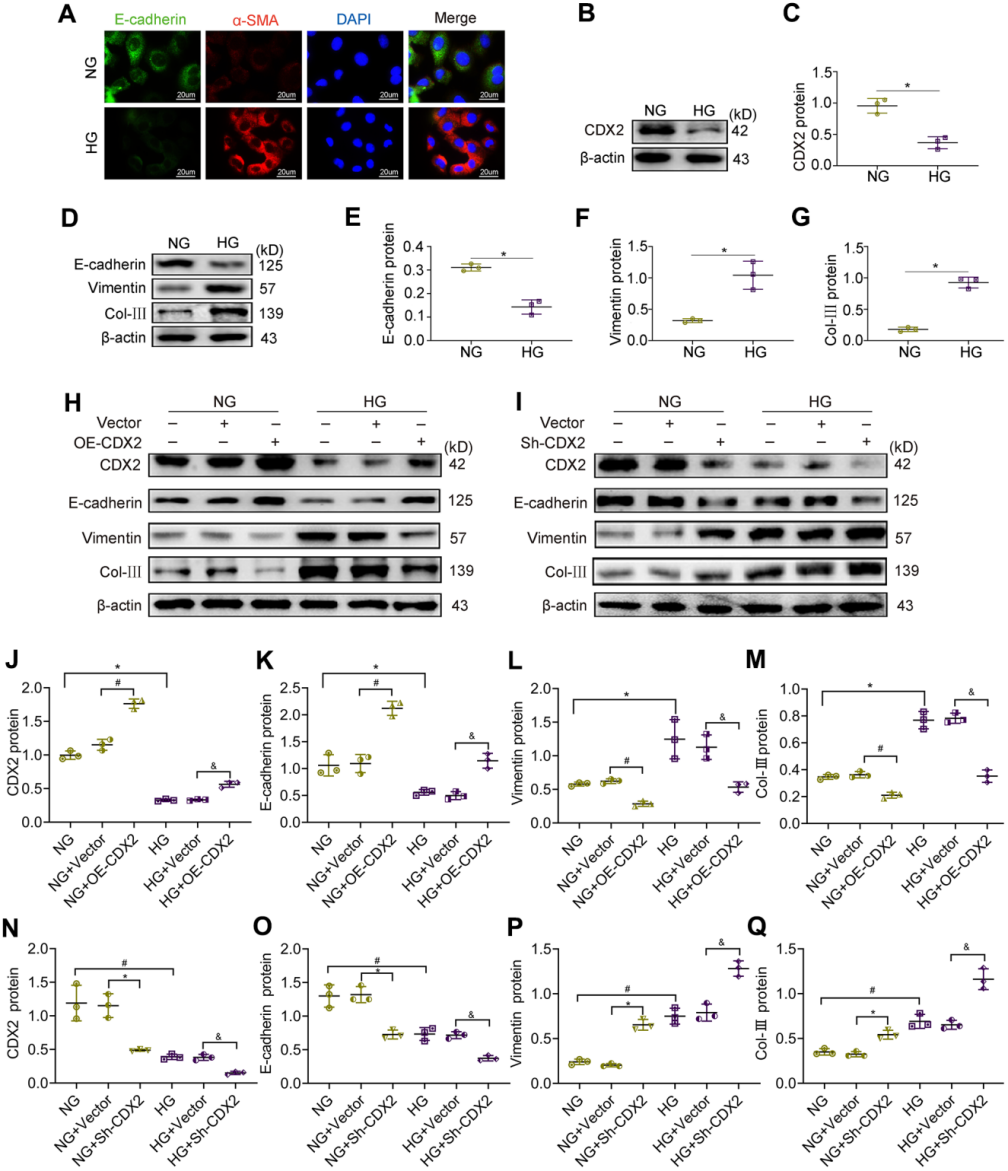
**CDX2 overexpression alleviates hyperglycemia-induced RTECs damage, and CDX2 knockdown aggravates the damage.** NRK-52E cells were administered NG (5.5 mM) and HG (25 mM) media for 48h, followed by analysis. (**A**) Immunofluorescence for E-cadherin and α-SMA detection in NRK-52E cells of the NG and HG groups, respectively (scale bar, 20μm). (**B**, **C**) Immunoblot bands of CDX2 (**B**) and quantitative data (**C**) in NRK-52E cells of the NG and HG groups. (**D**–**G**) Immunoblot bands of E-cadherin, Vimentin, and Col-III (**D**), and quantitative data (**E**–**G**) in NRK-52E cells of the NG and HG groups. H-Q Immunoblot bands of CDX2, E-cadherin, Vimentin, and Col-III in non-transfected (NG or HG treated) NRK-52E cells, and NRK-52E cells transfected with Vector (NG+Vector group, HG+Vector group), or CDX2-overexpressing (NG+OE-CDX2 group, HG+OE-CDX2 group) or CDX2-knockdown (NG+Sh-CDX2 group, HG+Sh-CDX2 group) (**H**, **I**); quantitative data are shown (**J**–**Q**). Data are mean±SD from three assays performed independently. n=3; ^*^P<0.05 versus NG group; ^#^*P*<0.05 versus NG+Vector group; ^&^*P*<0.05 versus HG+Vector group.

Hyperglycemia is the main feature of diabetes. To further assess CDX2’s role in DKD, NRK-52E cells were cultured with high glucose (HG) media to simulate renal tubular cells during DKD. Representative immunofluorescence micrographs showed that NRK-52E cells cultured under NG conditions featured abundant E-cadherin, an epithelial cell marker, which was decreased under HG conditions alongside the production of the mesenchymal-like protein α-SMA (Figure 3A). Cells in HG condition lost cell adhesion proteins, induced partial EMT and secreted collagen. In addition, CDX2 decreased with increasing glucose concentration (Supplementary Figure 2), CDX2 protein expression is negatively correlated with Snail expression, but positively correlated with E-cadherin expression (Supplementary Figure 3). To further investigate the relationship between CDX2 and cell phenotype, CDX2 was overexpressed or knockdown in cells exposed to NG and HG. Compared with control values, the protein levels of E-cadherin were increased while Vimentin and Col-III amounts were decreased in cells after CDX2 overexpression (Figure 3H, 3J–3M). However, after CDX2 silencing, E-cadherin protein amounts were decreased, while Vimentin and Col-III amounts were elevated in cells with cultured NG; these changes were further exacerbated in cells cultured with HG (Figure 3I, 3N–3Q). These findings indicated that CDX2 supplementation reversed hyperglycemia-induced loss of cell adhesion proteins and partial EMT in renal tubular epithelium, and suppression of CDX2 weakened cell junctions of renal tubular epithelial cells and induced partial EMT. Immunofluorescence micrographs showed thatCDX2 overexpression alleviates hyperglycemia-induced RTECs damage, and CDX2 knockdown induces the damages (Supplementary Figure 4), and overexpression of CDX2 *in vivo* can alleviate glomerular damage and renal tubular lesions during DKD (Supplementary Figure 5). These results confirmed that CDX2 inhibited high glucose-induced renal tubular injury by maintaining cell phenotype and inhibiting partial EMT.

### CFTR is regulated by CDX2 and decreased in DKD

**Figure 4:**
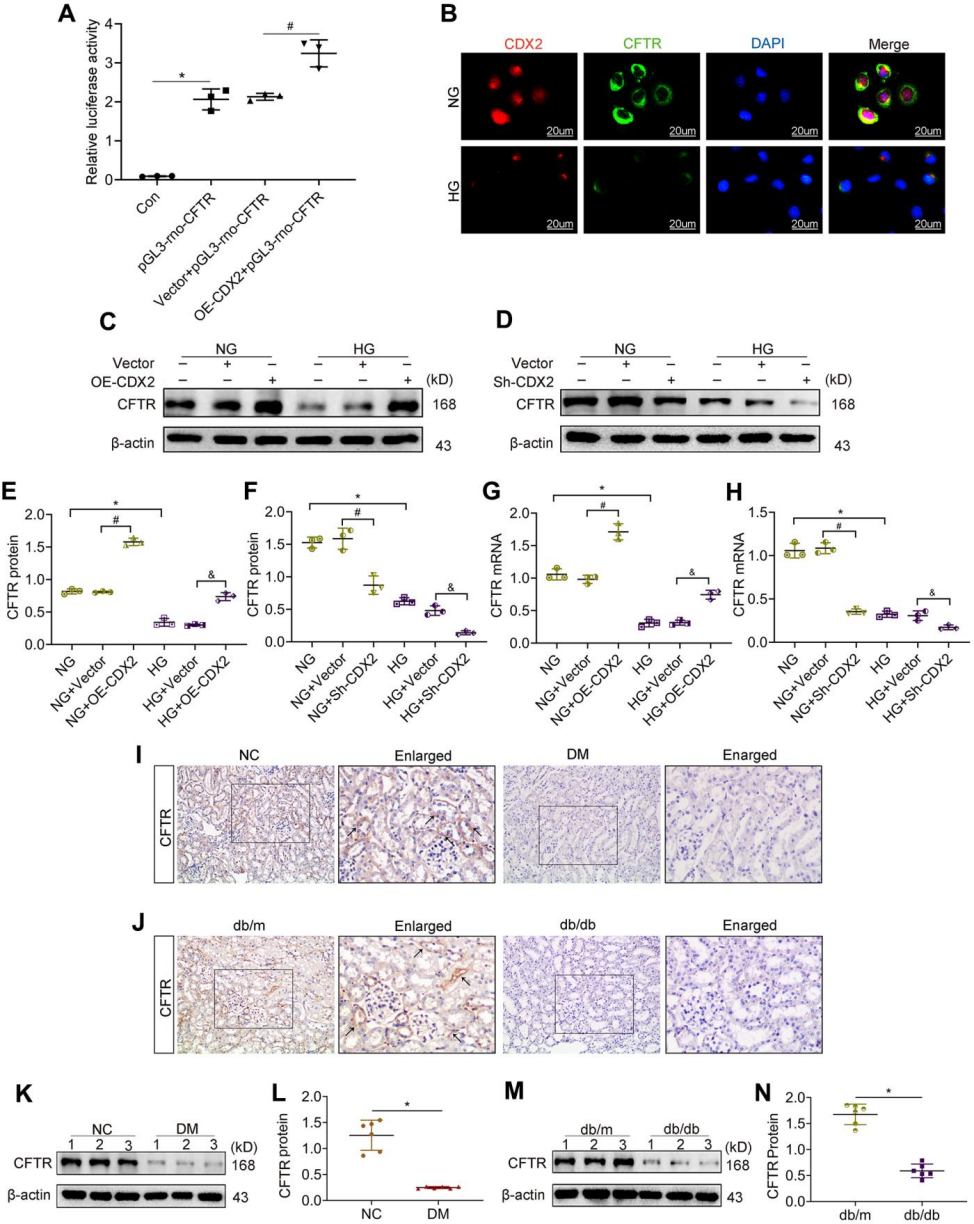
**CFTR is a downstream target gene of CDX2, and downregulated in DKD.** (**A**) CFTR promoter activity increased in CDX2-overexpressing NRK-52E cells. NRK-52E cells were administered non-transfected (Con)/pGL3-rno-CFTR-promoter plasmid (pGL3-rno-CFTR), or co-transfected with CDX2-overexpressing/Vector plasmid and pGL3-rno-CFTR-promoter plasmid. n=3; ^*^P<0.05 versus Con group; ^#^*P*<0.05 versus Vector + pGL3-rno-CFTR group. (**B**) Immunofluorescent staining of CDX2 and CFTR in NRK-52E cells of the NG and HG groups, respectively (scale bar, 20μm). C-H Western blot bands (**C**, **D**) and protein quantitation (**E**, **F**) of CFTR in non-transfected (NG or HG treated) NRK-52E cells, and NRK-52E cells transfected with Vector (NG+Vector, HG+Vector), CDX2-overexpressing (NG+OE-CDX2, HG+OE-CDX2) or CDX2-knockdown (NG+Sh-CDX2, HG+Sh-CDX2) plasmid, and mRNA levels (**G**, **H**). Data are mean±SD from three experiments performed independently. n=3; ^*^P<0.05 versus NG group; ^#^*P*<0.05 versus NG+Vector group; ^&^*P*<0.05 versus HG+Vector group. (**I**, **J**) Immunohistochemical staining of CFTR in T1D model mice and controls (**J**), T2D mice and controls (**J**). n=6; ^*^P<0.05 versus NC group or db/m group. (**K**, **L**) Western blot bands of CDX2 in T1D model mice and controls (**K**), and quantitative data (**L**). (**M**, **N**) Western blot bands of CFTR in T2D model mice and controls (**M**), and quantitative data (**N**). Data are mean±SD from three independent assays. n=6; ^*^P<0.05 versus NC group or db/m group.

As demonstrated above, CDX2 could represent a protective factor in renal tubular epithelium; however, the specific underlying mechanism is unclear. Studies have shown that CDX2 positively regulates CFTR by binding to *CFTR* intron elements in intestinal epithelial cells [19, 20]. CFTR is a chloride channel protein associated with kidney damage. Therefore, in order to explore the mechanism of CDX2 in renal tubular epithelium during DKD, we designed a luciferase reporter assay to test whether CFTR expression in NRK52-E cells is regulated by CDX2. We found that fluorescence signals were increased after CDX2 overexpression compared with the control group (Vector + pGL3-rno-CFTR group) (Figure 4A). This finding suggested that in NRK-52E cells, CDX2 enhanced CFTR transcription. *In vitro*, CDX2 was detected in the cytoplasm and nucleus, which was consistent with its localization in the kidney tissue (Figure 1A, 1C, 1K). Meanwhile, CFTR, a chloride channel protein, is localized in the cell membrane and cytoplasm, and both CDX2 and CFTR were downregulated after exposure to high glucose in renal tubular epithelial cells (Figure 4B). We further evaluated the effects of CDX2 on CFTR protein and transcription levels in NRK-52E cells. In comparison with control values, CFTR protein and mRNA amounts were increased after CDX2 overexpression, but decreased after CDX2 knockdown (Figure 4C–2H). *In vivo*, we found that CFTR was mainly expressed in the membrane and cytoplasm of renal tubular cells, and decreased in the kidneys of T1D and T2D animals (Figure 4I, 4J). Consistently, the protein levels of CFTR were reduced in the kidneys of both T1D and T2D animals (Figure 4K–4N). These results suggested that CDX2 regulated CFTR at the transcriptional level in RTEC, and both molecules were downregulated in DKD.

### CDX2 inhibits β-catenin activity by upregulating CFTR that binds to Dvl2

**Figure 5:**
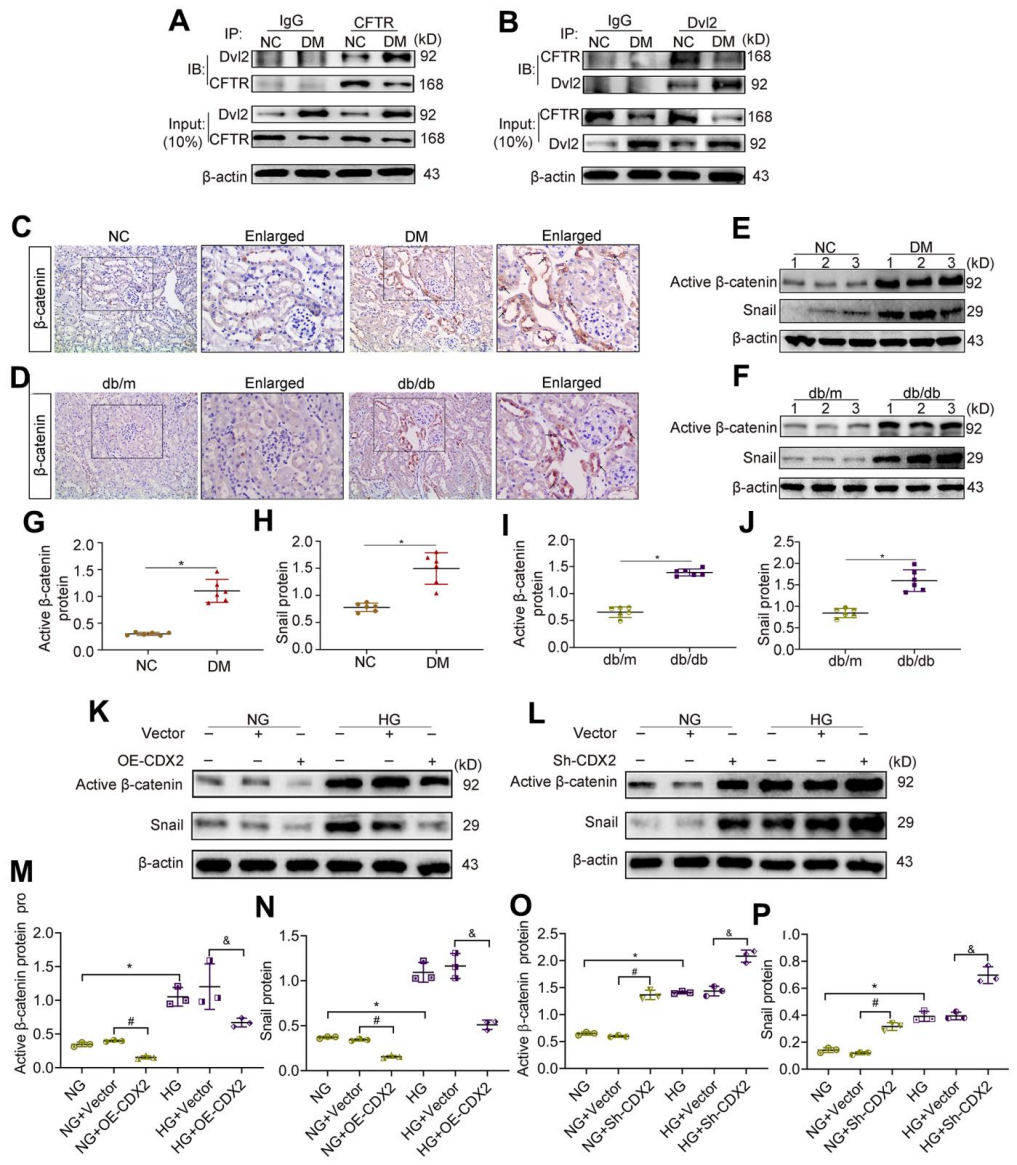
**Downregulation of CFTR activates β-catenin and causes renal fibrosis.** (**A**, **B**) Co-immunoprecipitation assay indicated that CFTR and Dvl2 interacted with each other *in vivo*. The Input group was a positive control group. In the CO-IP group, the kidney tissue lysates of T1D mice and controls were immunoprecipitated with IgG, anti-CFTR (**A**) or anti-Dvl2 (**B**) antibodies, and the resulting immunoprecipitates were blotted (IB) with anti-CFTR and anti-Dvl2 antibodies. The protein samples used for co-immunoprecipitation were normalized to β-actin. (**C**, **D**) Immunohistochemical staining of β-catenin in T1D model mice and controls (**C**), and T2D mice and controls (**D**). Positive staining (black arrow) (magnification, ×200); enlarged box area (magnification, ×400). (**E**–**J**) Western blot bands of Activated β-catenin and Snail in T1D model mice and controls (**E**), and T2D model mice and controls (**F**); quantitative data are shown (**G**–**J**). n=6; ^*^P<0.05 versus NC group or db/m group. (**K**–**P**) Western blot bands (**K**, **L**) and quantitative data (**M**–**P**) of activated β-catenin and Snail in non-transfected (NG group, HG group) NRK-52E cells, and NRK-52E cells transfected with Vector (NG+Vector group or HG+Vector group), or CDX2-overexpressing (NG+OE-CDX2 group or HG+OE-CDX2 group) or CDX2-knockdown (NG+Sh-CDX2 group or HG+Sh-CDX2 group) plasmid. Data are mean±SD from three experiments performed independently. n=3; ^*^P<0.05 versus NG group; ^#^*P*<0.05 versus NG+Vector group; ^&^*P*<0.05 versus HG+Vector group.

CFTR inhibits UUO-induced renal fibrosis by interfering with β-catenin activation [14]. The cytoplasmic protein Dvl, a key adapter protein of Wnt/β-catenin signaling, possesses a PDZ domain capable of binding to CFTR. We found that the protein levels of CFTR were decreased while Dvl2 amounts were elevated in DM animals in the Input group, in comparison with the NC group. Dvl2 was detected in lysates immunoprecipitated with anti-CFTR antibodies (Figure 5A). Conversely, CFTR was detected in lysates immunoprecipitated with anti-Dvl2 antibodies (Figure 5B). In the kidneys of T1D and T2D, β-catenin amounts were increased, and the protein was translocated into the nucleus (Figure 5C, 5D). The protein amounts of activated β-catenin and its target Snail increased (Figure 5E–5J). β-catenin activation and nuclear transfer promoted Snail transcription. Snail causes renal tubular-interstitial fibrosis in DKD by inhibiting E-cadherin and inducing partial EMT [15, 16]. After CDX2 overexpression, Active β-catenin (activated β-catenin) and Snail amounts were reduced in NRK-52E cells exposed to normal or high-glucose (Figure 5K, 5M, 5N). However, CDX2 downregulation elevated activated β-catenin and Snail amounts (Figure 5L–5P). These results suggested that CFTR suppressed β-catenin activation by binding to Dvl2 to stop signal transmission in DKD. CDX2 inhibited the increase of activated β-catenin and Snail to suppress hyperglycemia-associated RTECs injury and renal tubular-interstitial fibrosis, and it’s effects may rely on CFTR upregulation.

### CDX2 prevents hyperglycemia-associated renal tubular lesions by positively regulating CFTR to suppress β-catenin activation

**Figure 6:**
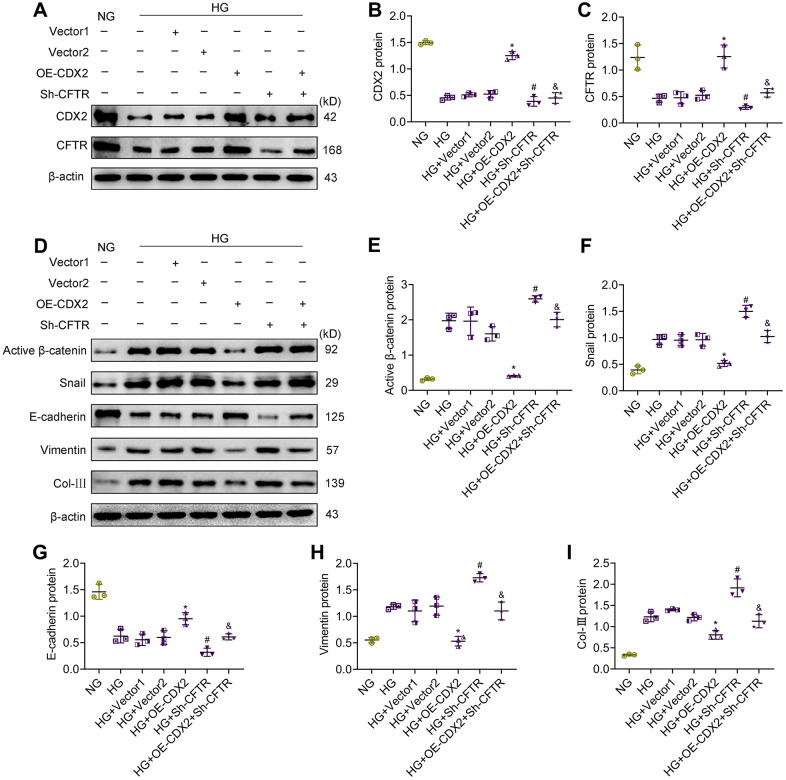
**CFTR knockdown abolishes the effects of CDX2 on resisting hyperglycemia-induced RTECs injury.** NRK-52E cells were, respectively, non-transfected (NG group or HG group), and transfected with CDX2-overexpressing / Vector1 (HG+OE-CDX2 group / HG+Vector1 group) or CFTR-knockdown / Vector2 plasmid (HG+Sh-CFTR group / HG+Vector2 group), or co-transfected CDX2-overexpressing + CFTR-knockdown plasmid (HG+OE-CDX2+Sh-CFTR group). (**A**–**C**) Immunoblot bands of CDX2 and CFTR, and quantitative data (**B**, **C**). (**D**–**I**) Immunoblot bands of activated β-catenin, Snail, E-cadherin, Vimentin, and Col-III; quantitative data are shown (**E**–**I**). Data are mean±SD from three experiments performed independently. n=3; ^*^P<0.05 versus HG+Vector1 group; ^#^*P*<0.05 versus HG+Vector2 group; ^&^*P*<0.05 versus HG+OE-CDX2 group.

In order to confirm that CDX2 inhibits β-catenin activation by upregulating CFTR, reducing renal tubular epithelial fibrosis, we knocked down CFTR while overexpressing CDX2 in NRK-52E cells under HG conditions. The results showed that CDX2 and CFTR protein amounts were reduced after stimulation with HG, while activated β-catenin and Snail amounts were elevated; cell junction was reduced but renal tubular lesions was induced. After CDX2 overexpression under HG conditions, the expression of CFTR increased, and Wnt/β-catenin signaling and renal tubular lesions were inhibited. However, after CFTR silencing under HG conditions, there was no change in CDX2 expression. After CDX2 overexpression and knockdown of CFTR under HG conditions, CDX2 cannot present the suppression of Wnt/β-catenin signaling to inhibit hyperglycemia-associated renal tubular lesions (Figure 6). These evidences revealed that CDX2 interfered with β-catenin activation by positively regulating CFTR, ultimately inhibiting hyperglycemia-associated renal tubular lesions.

## DISCUSSION

Cell junction proteins are key to maintaining the molecular structure and selective barrier function of renal tubules, whose dysfunction allows macromolecular protein antigens and toxic substances in the urine to cross the epithelial barrier to the sub-epithelial area, inducing tubular and interstitial inflammation as well as fibrosis [4]. Partial EMT [21] features abnormally differentiated RTECs, with loss of epithelial cell biomarkers (e.g., the cell adhesion protein E-cadherin) and increased mesenchymal biomarkers (e.g., α-SMA and Vimentin), as well as large amounts of growth factors such as TGF-β [22], to activate fibroblasts and induce kidney fibrosis.

In recent years, it was found that CDX2 suppression is associated with tumor invasion and migration, and the underlying mechanism involves promoting cell junction protein expression and inhibiting EMT [12]. CDX2 is a gut-specific tail-type homeobox nuclear transcription factor that mainly induces intestinal epithelium formation and maintains its normal differentiation [23]. The CDX2 protein comprises 311 amino acids, binds to the corresponding region of DNA by a helix-loop-helix interactions, and plays critical roles in cell proliferation, differentiation, adhesion, and apoptosis by regulating DNA expression as a transcription factor [24, 25]. Previous research assessing CDX2 has basically focused on gastrointestinal diseases. However, the pathological role of CDX2 remains controversial, and inconsistencies may be related to tumor location and type [7–10]. The present study demonstrated that CDX2 was abundant in non-diseased kidneys of mice and humans, and mostly detected in renal tubules. In the kidney tissue of T1D, T2D mice and DKD patients, CDX2 was decreased at the transcription and protein levels in kidney tissues, and kidney CDX2 is downregulated in IgA nephropathy and in UUO. Meanwhile, renal CDX2 amounts were negatively correlated with the degree of renal fibrosis. This finding suggested that reduction of CDX2 might represent an important factor involved in the formation of renal tubular lesions in DKD.

In gastric cancer, CDX2 inhibits cell invasion and metastasis by upregulating the adhesion protein E-cadherin and inhibiting EMT [12]. Similarly, we found that CDX2 could induce E-cadherin production in NRK-52E cells. E-cadherin represents a transmembrane glycoprotein termed adhesion junction, and is important in maintaining the normal structure and function of normal epithelial tissues [26]. In DKD, loss of E-cadherin in RTECs weakened intercellular adhesion; therefore, RTECs are detached, which impairs renal tubular barrier function, resulting in tubular-interstitial inflammation and fibrosis [4, 27]. The phenotype of RTECs and epithelial integrity are closely associated with the normal function of renal tubules [26]. EMT is considered a sign of tumor transformation [28]. In renal fibrosis, EMT is commonly described as a process in which epithelial cells completely undergo conversion into fibroblasts, but increasing evidence has confirmed that instead of direct transformation to myofibroblasts, renal epithelial cells remain in the tubules, showing partial EMT, which promotes tubulointerstitial fibrosis and inflammation [21, 29–31]. Partial EMT was detected in DKD, downregulation of molecular epithelial markers (e.g. E-cadherin) co-occurring with upregulated mesenchymal biomarkers (Vimentin and Col-III). Most importantly, our data showed that CDX2 inhibited partial EMT in RTECs. *In vitro*, both CDX2 silencing and HG treatment reduced E-cadherin amounts and induced partial EMT in RTECs, while its overexpression of CDX2 inhibited high glucose-mediated E-cadherin loss and partial EMT. *In vivo*, overexpression CDX2 improved renal function and renal fibrosis in T1D. These data indicated that CDX2 prevents renal tubular lesions in DKD by promoting cell junction protein formation and inhibiting partial EMT.

CFTR is broadly expressed in mammalian kidneys. Its dysfunction causes cystic fibrosis (CF), which results in lifetime microalbuminuria in >6% of cases [32], indicating that CFTR defect is associated with kidney damage. MatInspector (https://www.genomatix.de/) predictive analysis revealed that CDX2 interacts with CFTR enhancer, whose activity is reduced by more than 60% after mutating these sites [19]. In the context of intestinal epithelial cells, CDX2 can enhance CFTR gene expression [19, 20]. In this study, we demonstrated that CDX2 upregulated CFTR in NRK-52E cells by luciferase reporter assy. Moreover, CDX2 increased CFTR protein amounts in RTECs. We assessed CFTR in the renal tissue, and found that CFTR was also mainly distributed in the cell membrane and cytoplasm of renal tubules, showing decreased levels in DKD. Downregulation of CFTR in DKD may be related to reduced CDX2 expression.

Recent studies have reported that CFTR suppresses Wnt/β-catenin signaling and inhibits UUO-induced kidney fibrosis [14]. Wnt/β-catenin signaling represents a critical pathway that has been evolutionarily conserved to regulate kidney development, and its aberrant induction triggers widespread changes in the kidney, including repair and fibrosis of RTECs, podocyte injury, podocyte dedifferentiation and mesangium fibrosis, to cause DKD [33, 34]. The Wnt/β-catenin signaling pathway is relatively silent in non-diseased human kidneys, and cytosolic β-catenin is phosphorylated by “the degradation complex” and eventually degraded by the proteasome. Increased amounts of activated β-catenin (undegraded β-catenin) and nuclear β-catenin are usually considered an activation marker of Wnt/β-catenin signaling [35, 36]. Dvl represents a key component of β-catenin’s signal transmission, with Dvl2 being the most abundant of its three homologous genes. In DKD, Dvl is recruited by Frizzled (Fz) and transmits signals through its PDZ domain to connect Wnt receptors and downstream signaling components, causing the disintegration of the degradation complex [37]. Then, β-catenin escapes the degradation and accumulates in the cytoplasm in order to translocate into the nucleus and interact with transcription factors such as lymphoid enhancer factor/T cell factor (LEF/TCF), promoting the transcription of genes (e.g., Snail and TWIST) and driving renal tubule-interstitial fibrosis [34, 38]. Interestingly, the C terminus of CFTR contains a PDZ-binding domain [39], indicating that this protein can bind to Dvl. This was confirmed in the renal tissue; in normal kidney or DKD, CFTR can interact with Dvl2, but CFTR-Dvl2 complex amounts are low due to reduced CFTR amounts and increased Dvl2 during DKD; in this case, unbound Dvl2 may act as a transmission molecule for the Wnt signaling pathway, eventually activating β-catenin. This is consistent with findings by Jie Ting Zhang in UUO [14].

Snail family zinc finger 1 (SNAI1/Snail) represents a transcription factor produced in various settings of kidney injury; SNAI1 modulates multiple biological events causing renal fibrogenesis, including destruction of cell adhesion, partial EMT of RTECs and renal interstitial inflammation [16, 40]. We demonstrated that in DKD kidney tissue, activated (undegraded) β-catenin was increased; in addition, β-catenin was translocated into the nucleus, and its downstream protein Snail was upregulated. What’s more, we found that CDX2 overexpression inhibited high glucose-induced β-catenin activation and snail expression, while CDX2 knockdown promotes β-catenin activation and snail expression. This effect of CDX2 on the Wnt/β-catenin signaling may be related to CFTR.

To further explore the regulatory associations and roles of CDX2, CFTR and Wnt/β-catenin signaling in DKD, CDX2 was overexpressed while knocking down CFTR in NRK-52E cells. We found that the inhibitory effect of CDX2 on high glucose-mediated renal tubular injury was significantly reduced after CFTR silencing, with increased β-catenin and Snail amounts. These findings further confirmed that CDX2 exerts protective effects in DKD by regulating CFTR to suppress β-catenin activation.

In summary, CDX2 plays a protective role in DKD pathogenesis by promoting cell junction formation and inhibiting partial EMT in renal tubular epithelial cells to counter tubulointerstitial fibrosis. Its specific mechanism involves CDX2 inhibition of β-catenin activation and nuclear transfer by upregulating CFTR. These findings are important because prevention and treatment of tubular damage is essential for early prevention in DKD, and CDX2 can maintain the structure and function of tubular epithelial cells.

## MATERIALS AND METHODS

### Human kidney samples

Renal samples were obtained from 6 individuals diagnosed with DKD (5 males and 1 female; aged 48.83±7.80 years) at the Affiliated Hospital of Guizhou Medical University. In 6 patients (6 males; aged 29.33±10.23 years) showing kidney lesions other than tumors (e.g., renal trauma), renal tissues were pathologically confirmed to be normal, and used as normal controls (Patient information is shown in Supplementary Table 1). The current study had approval from the Ethics Committee of the Affiliated Hospital of Guizhou Medical University (No.112).

### Animal models

All mice were provided by Model Animal Research Center of Nanjing University (MARC, China). Male db/db mice generated in C57BLKS/JNju background (6 weeks old) were assessed as type 2 diabetes (T2D) model mice (db/db). Meanwhile, age-matched male C57BLKS/JNju mice, also from MARC, served as the control group (db/m). Both mouse groups were submitted to euthanasia at 18 weeks of age. Twelve C57BL/6J mice (8 weeks old) were randomized into the T1D (DM, n=6) and normal control (NC, n=6) groups after one week of adaptive feeding. The DM group was intraperitoneally treated with 55 mg/kg streptozotocin (STZ, in sterile citrate buffer [pH 4.5]; Sigma) for 5 days, while the NC group was administered the same amount of vehicle. After 2 days, fasting blood glucose was measured continuously for 3 times. Successful modeling was considered for mice with blood glucose levels of 16.7 mmol/L or more. Animal housing was performed at 22±2° C under a 12h-12h light/dark cycle, with water and food freely available. Mice were killed at 16 weeks of age (6-weeks after STZ injection). Before euthanasia, 24-h urine specimens from all mice were obtained using metabolic cages, and total urine volumes were assessed. Mice underwent 3–4h fasting prior to anesthesia with diethyl ether, followed by femoral artery puncture for collecting blood samples employed for serum preparation. Urine and serum specimens were kept at −20° C for biochemical assays. After sacrifice, both kidneys from each animal were extracted, with one submitted to 4% formalin fixation for histological analysis and the other immediately placed in liquid nitrogen and kept at −80° C for molecular studies.

All assays involving animals had approval from the Animal Experimental Ethics Committee of Guizhou Medical University (No.2000032).

### Cell culture and transfection

Normal rat kidney tubular epithelial NRK-52E cells (Jennio Biotech, China) underwent maintenance culture in Dulbecco's modified Eagle's medium (DMEM; Gibco, USA) supplemented with 10% fetal bovine serum (FBS; Gibco) and 5.5 mM glucose, in an incubator containing 5% CO2 and maintained at 37° C.

Cell growth was then performed in normal (NG; 5.5 mM) and high (HG; 25 mM) glucose media, separately, supplemented with 2% FBS. NRK-52E cells were transiently transfected with Lipofectamine 3000 (Invitrogen, USA) based on the kit’s protocol. All plasmids (*CDX2* plasmid, *pCMVPuro01-CDX2*; *CDX2 shRNA* plasmid, *CDX2-shRNA-GP*; *CFTR shRNA* plasmid, *CFTR-shRNA-GP*) were purchased from Longqian Biotech (China).

### Biochemical assays

Serum glucose and urine microalbumin amounts were assessed on a Beckman Instruments 1650 automated bioanalyzer (Beckman Instruments, USA). 24 hour urine microalbumin (mg/24h) was assessed as follows: microalbumin (mg/ml) × urine volume (ml)/24h.

### Histological analysis and immunohistochemistry

Paraffin-embedding of kidney specimens and sectioning were performed via standardized procedures. The resulting sections underwent staining with periodic acid-Schiff (G1360; Solarbio, China) and Sirius red staining (BB-44333; BestBio, China) reagents according to respective recommended protocols. Immunohistochemical staining was carried out using a Two Step Immunoassay assay kit (ZSBIO, China), as directed by the manufacturer. The antibodies used included rabbit monoclonal anti-CDX2 (YM3057; Immunoway Bio, USA), mouse monoclonal anti-CFTR (sc-376683; Santa Cruz, USA), and rabbit polyclonal anti-β-catenin (bs-1165R; Bioss, Beijing, China). Areas of positive staining were quantified by ImageJ in 6 random fields (200×) per sample, with three individuals assessed in each group.

### Immunofluorescence

Kidney specimens, stored at -80° C, were thawed at room temperature, and kidney cryosections were performed according to standard procedures. NRK-52E cells grown on coverslips underwent 4% formalin fixation for 1h. Upon blocking with 5% bovine serum albumin (BSA) at ambient, anti-E-cadherin (14472; Cell Signaling Technology, USA), anti-α-SMA (55135; Proteintech, China) and anti-collagen type III (22734; Proteintech) primary antibodies were added for incubation at 4° C overnight. This was followed by staining with FITC– or Cy3–linked secondary antibodies (Proteintech). DAPI (4′,6-diamidino-2-phenylindole) counterstaining was carried out, and samples were assessed under a Leica DM4000B fluorescence microscope (Leica, Germany).

### Western blot

Cell or tissue lysis was carried out with RIPA buffer (R0020; Solarbio, China), and total protein amounts were determined with the BCA kit (PC0020; Solarbio). After addition of the corresponding loading buffer (P1040 or P1019; Solarbio), the mixture underwent a 10-min boiling step. Equal amounts of total protein were resolved by SDS-PAGE and electro-transferred onto PVDF compound membranes (Millipore, USA) treated with methanol. Upon blocking with 5% non-fat milk, the membranes underwent overnight incubation (4° C) with primary antibodies raised against CDX2 (60243; Proteintech, 1:1000), CFTR (2784; Abcam, 1:1000), active β-catenin (8814; CST, 1:1000), Snail (3879; CST, 1:1000), E-cadherin (14472; CST, 1:1000), Vimentin (5741;CST, 1:1000), Collagen type III (Col-III; 22734; Proteintech, 1:1000, Fibronectin (2413; Abcam, 1:1000) and β-actin (Pumei, China, 1:4000), respectively. Then, secondary antibodies were added at ambient for 1h. Finally, the ECL solution was added, and a Bio-Rad gel imaging system (Bio-Rad, USA) was employed for analysis.

### Real time-quantitative PCR (qRT-PCR)

Total RNA was purified from tissue and cell specimens with TRIzol reagent (Invitrogen) as directed by the manufacturer. Reverse transcription was carried out with reverse transcription kit (K1622; Thermo Scientific, USA), and the Real-Time PCR kit (FP209-02; TIANGEN, China) was used for qRT-PCR on an ABI 7500-Fast Real-Time PCR System (Applied Biosystems, USA). The data were normalized to β-actin expression, and analyzed by the 2^−ΔΔCt^ method. Primers are described in Table 1.

**Table 1:** Primers used in qRT-PCR.

**Gene**	**Sequence**
*CDX2* (NM 007673.3)-mouse	Forward: 5’-CCAAGTGAAAACCAGGACAAAA-3’
	Reverse: 5’-TGCTGCTTCTTCTTGATTTTCC-3’
*CFTR* (NM 007673.3)-mouse	Forward: 5’-AAAAGAATCCCCAGCTTATCCA-3’
	Reverse: 5’-TTGGTGACTTCCCCTAGGTATA-3’
*β-actin* (NM 007393.5)-mouse	Forward: 5’-CTACCTCATGAAGATCCTGACC-3’
	Reverse: 5’-CACAGCTTCTCTTTGATGTCAC-3’
*CDX2* (NM 023963.1)-Rat	Forward: 5’-AGCGGCTGGAGCTGGAGAAG -3’
	Reverse: 5’-TGCTGCTGCTGCTGCTGTTG-3’
*CFTR* (NM 031506.1)-Rat	Forward: 5’-CGCTGGTTGCACAGTAGTCCTC-3’
	Reverse: 5’-AGGGCTCGCTGGAAGACACTC-3’
*β-actin* (NM 031144.3)-Rat	Forward: 5’-CAGCCTTCCTTCCTGGGTATG -3’
	Reverse: 5’-AGGGTGTAAAACGCAGCTCA-3’

### Co-immunoprecipitation

The tissue lysis method was the same as described for Western blot. The lysate was incubated with immunoglobulin G (IgG; negative control), and anti-CFTR (2784; Abcam) or Dvl2 (3224; CST) antibodies, respectively. Dynabeads^TM^ Protein G Immunoprecipitation Kit (10007D; Thermo Scientific) was employed for sample precession, as described by the manufacturer.

### Luciferase reporter assay

The CFTR-promoter luciferase reporter was constructed by Longqian Biotech (China). Actively growing NRK52E cells were trypsinized and seeded in 24-well plates at a suitable density for routine culture. After 24h, transfection was carried out with Lipofectamine 3000 (Invitrogen) as directed by the manufacturer for 48h. This was followed by cell lysis and sample analysis with a Dual-Luciferase Reporter Assay System (E1960; Promega, USA). Renilla and Firefly luciferase activities were read, and the ratio of Renilla luciferase activity to that of Firefly luciferase was derived. Triplicate experiments were repeated 3 times independently.

### Co-immunoprecipitation

The tissue lysis method was the same as described for Western blot. The lysate was incubated with immunoglobulin G (IgG; negative control), and anti-CFTR (2784; Abcam) or Dvl2 (3224; CST) antibodies, respectively. Dynabeads^TM^ Protein G Immunoprecipitation Kit (10007D; Thermo scientific) was employed for sample precession, as described by the manufacturer.

### Luciferase reporter assay

The CFTR-promoter luciferase reporter was constructed by Longqian Biotech (China). Actively growing NRK52E cells were trypsinized and seeded in 24-well plates at a suitable density for routine culture. After 24h, transfection was carried out with Lipofectamine 3000 (Invitrogen) as directed by the manufacturer for 48h. This was followed by cell lysis and sample analysis with a Dual-Luciferase Reporter Assay System (E1960; Promega, USA). Renilla and Firefly luciferase activities were read, and the ratio of Renilla luciferase activity to that of Firefly luciferase was derived. Triplicate experiments were repeated 3 times independently.

### Statistical analysis

Assays were performed at least 3 times independently, and animal experiments had 6 samples per group. Data are mean±standard deviation (SD). Unpaired Student t-test and one-way analysis of variance (ANOVA) were carried out for group pair and multiple group comparisons, respectively. Spearman (nonparametric) correlation analysis was performed to evaluate the association of CDX2 expression with pathology in DKD. SPSS 22 was used for data analysis. *P*<0.05 indicated statistical significance.

## Supplementary Material

Supplementary Figures

Supplementary Table 1
