# Supplementary material for: The role of CDX2 in renal tubular lesions during diabetic kidney disease
Source: Aging (Albany NY). 2021 Feb 17;13(5):6782–803. doi: 10.18632/aging.202537 (PMC7993706; doi:10.18632/aging.202537)
Supplement: Supplementary Figures [file aging-13-202537-s001.pdf]

SUPPLEMENTARY FIGURES

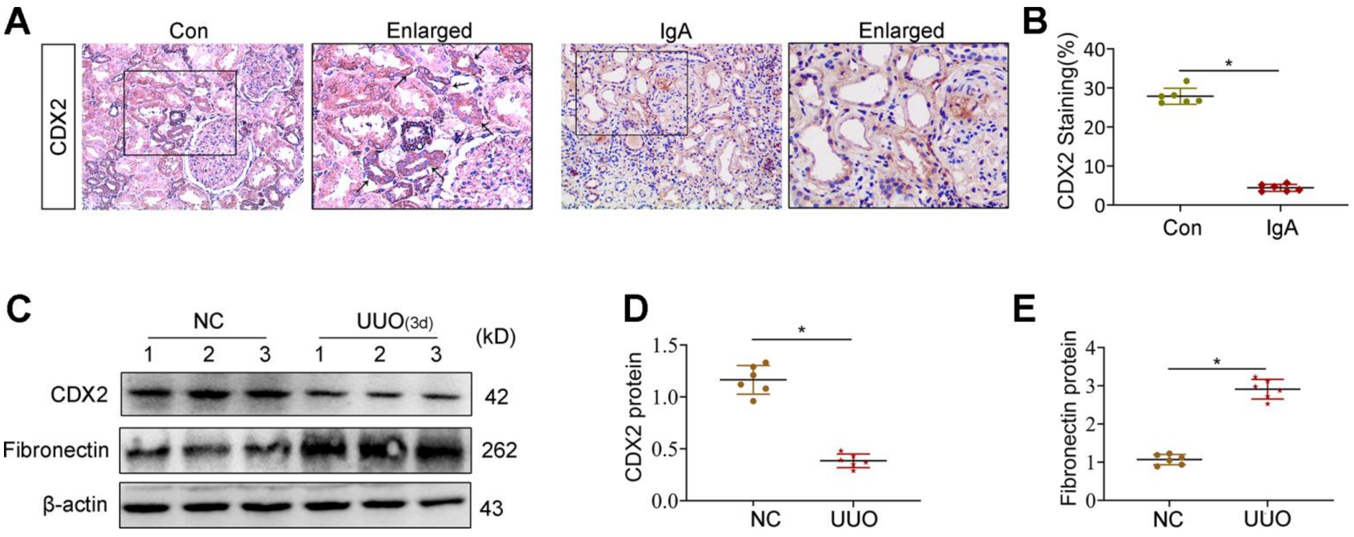

Supplementary Figure 1. Kidney CDX2 is downregulated in IgA nephropathy and in UUO. (**A**, **B**) Immunohistochemical staining (**A**) and quantitation (**B**) for CDX2 in IgA nephropathy patients kidney tissues and controls. CDX2 is expressed in the cytoplasm and nucleus of renal tubular epithelial cells in the renal cortex (magnification, ×200); enlarged box area (magnification, ×400). Immunohistochemical-positive staining density of CDX2 was analyzed in each group from 6 random fields (200×).All data are mean±SD from three independent experiments. *n*=6; **P*<0.05 versus Con group. (**C**–**E**) The mouse kidneys were harvested 3 days after unilateral ureteral obstruction (UUO)(*n* = 6 per group).Immunoblot (**C**) and quantitation (**D**, **E**) for CDX2 and Fibronectinin UUOmice and controls. All data are mean±SD from three independent experiments. *n*=6; **P*<0.05 versus NC group.

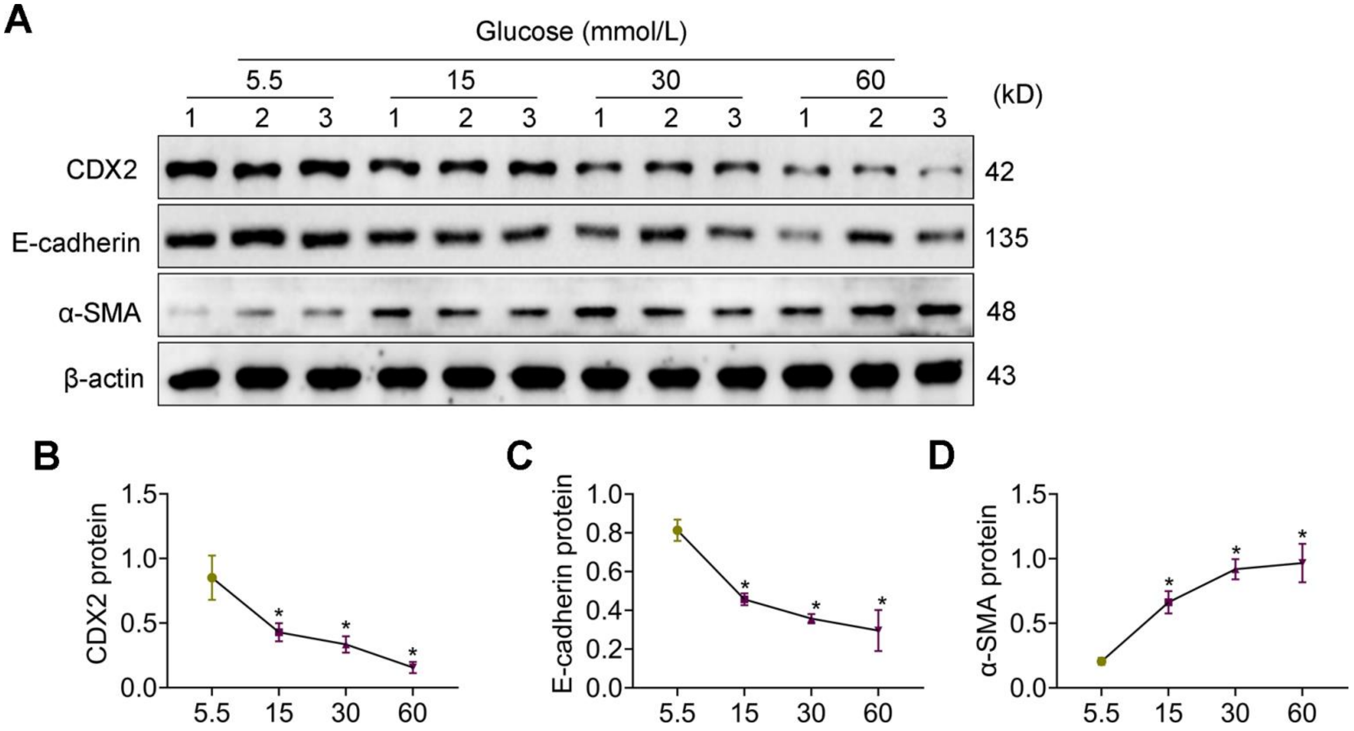

Supplementary Figure 2. The expression of CDX2 decreases with increasing glucose concentration. (**A**, **B**) The protein expression of CDX2 in NRK-52 cells cultured at a glucose concentration of 5.5-60mmol/L 48h was detected by Western blot. Immunoblot (**A**) and quantitation for CDX2 (**B**), E-cadherin (**C**) and α-SMA (**D**). All data are mean±SD from three independent experiments. n=3; **P*<0.05 versus 5.5 mmol/L group.

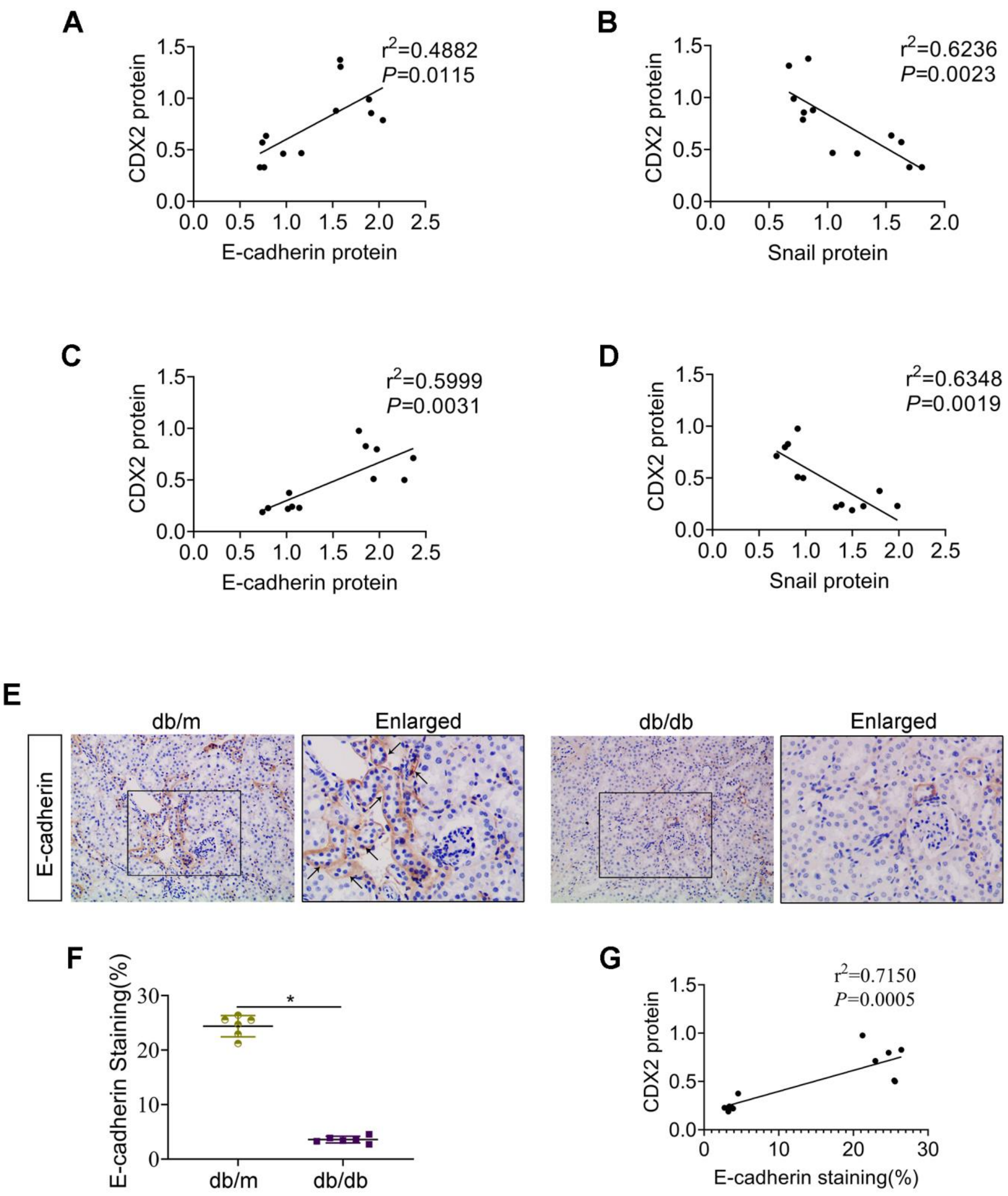

Supplementary Figure 3. CDX2 protein expression is negatively correlated with Snail expression, but positively correlated with E-cadherin expression. (**A**, **B**) Correlation of CDX2 protein with E-cadherin protein (A,*r*= 0.4882; *P* = 0.0115) and Snail protein (B, r= 0.6236; P= 0.0023) in the kidneys of T1D mice and controls. (**C**, **D**) Correlation of CDX2 protein with E-cadherin protein (C,*r*= 0.5999; *P* = 0.0031) and Snail protein (**D**, r= 0.6348; P= 0.0019) in the kidneys of T2D mice and controls. (**E**–**G**) Immunohistochemical staining (**E**) and quantitative analysis (**F**) of E-cadherin in T2D mice and controls. E-cadherin is expressed in the cytomembrane of renal tubular epithelial cells in the renal cortex (magnification, ×200); enlarged box area (magnification, ×400). Correlation of CDX2 protein with E-cadherin staining (**G**,*r*= 0.7150; *P* = 0.0005) in T2D mice kidney tissues and controls. All data are mean±SD from three independent experiments. *n*=6; **P*<0.05 versus db/m group.

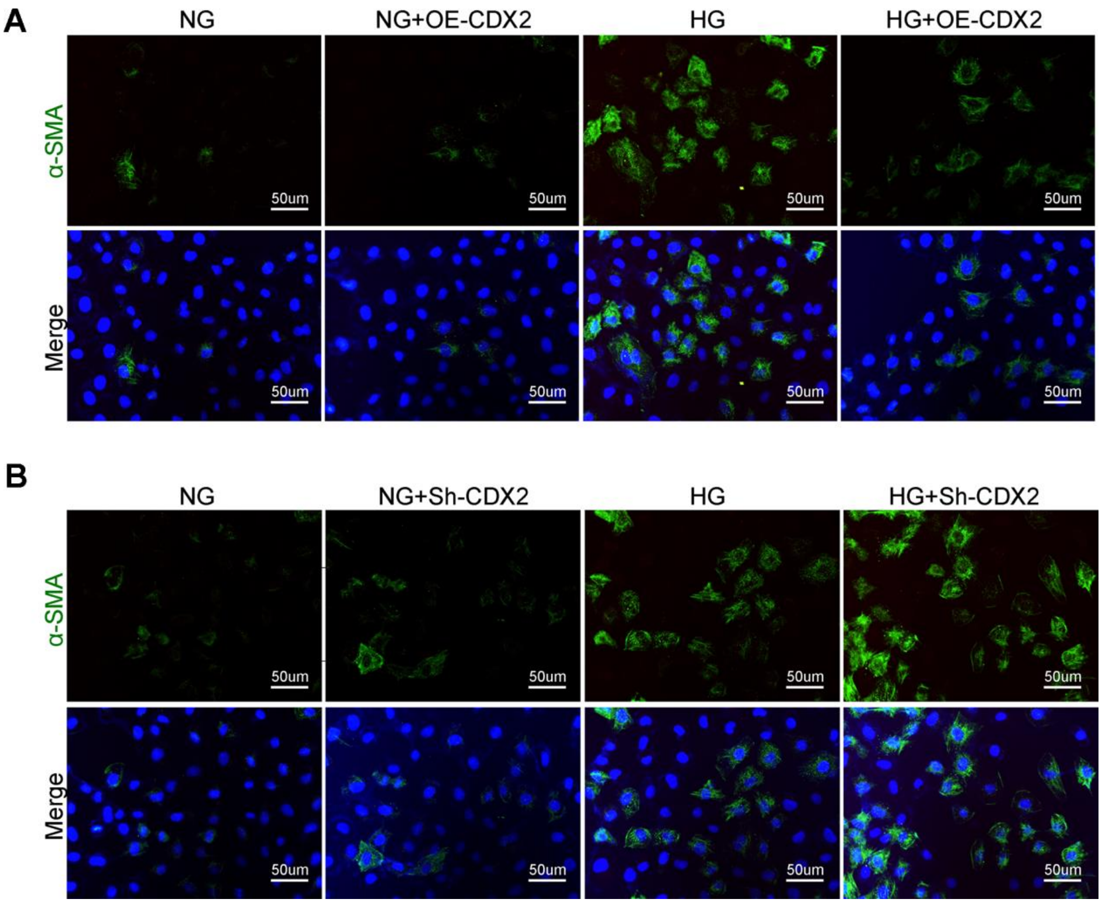

Supplementary Figure 4. CDX2 overexpression alleviates hyperglycemia-induced RTECs damage, and CDX2 knockdown induces the damage. Immunofluorescence of α-SMA in non-transfected (NG or HG treated) NRK-52E cells, and NRK-52E cells transfected with CDX2-overexpressing (NG+OE-CDX2 group, HG+OE-CDX2 group) or CDX2-knockdown (NG+Sh-CDX2 group, HG+Sh-CDX2 group), respectively (scale bar, 20μm). CDX2 overexpression alleviates hyperglycemia-induced RTECs damage.CDX2 overexpression inhibits the increase in α-SMA expression induced by high glucose, thereby inhibiting partial EMT of RTECs (**A**). CDX2 overexpression alleviates hyperglycemia-induced RTECs damage. CDX2 knockdown induces the damage of RTECs, and aggravates partial EMT of RTECs under high glucose condition (**B**). Data from three experiments performed independently.

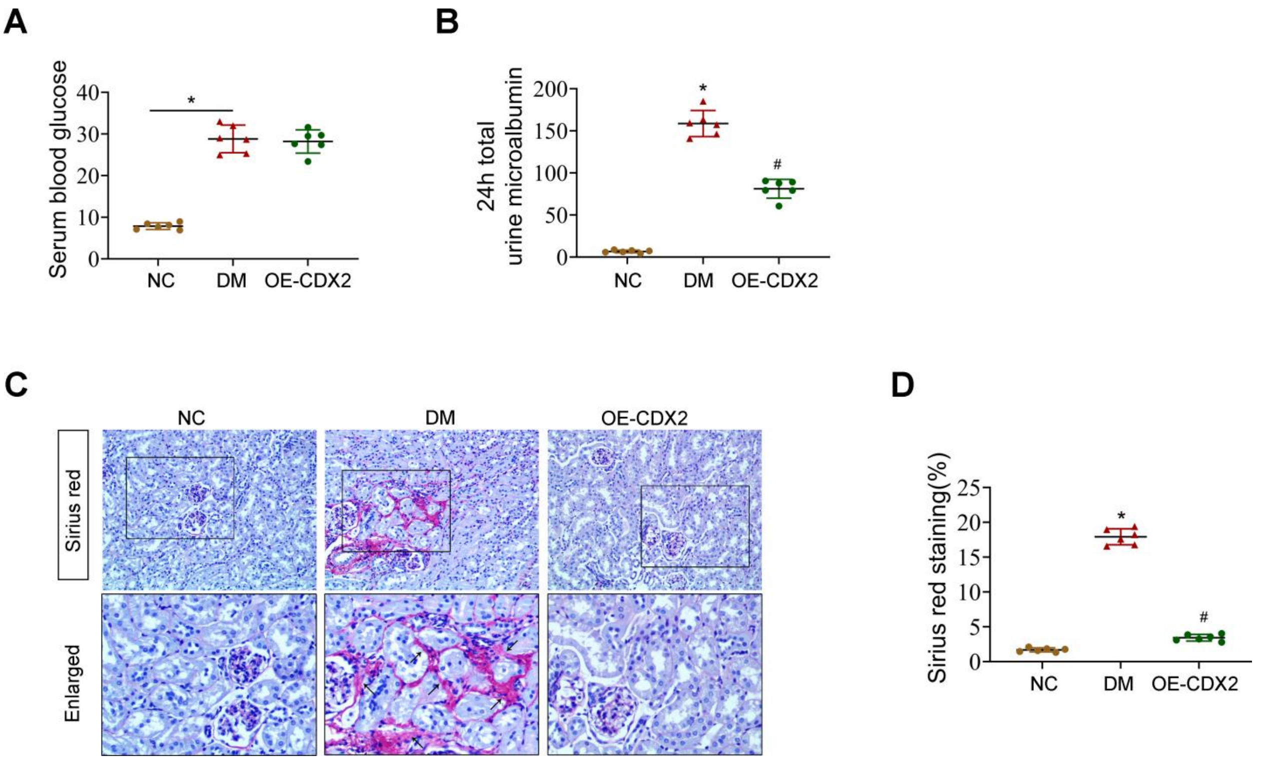

Supplementary Figure 5. CDX2 can improve renal fibrosis during DKD. After successfully replicating the T1D model by intraperitoneal injection of STZ, the mice were injected with 15 ug (diluted with Ringer's solution) through the tail vein, and were continuously injected for 6 weeks once a week. Mice were submitted to euthanasia at 14 weeks of age (*n*= 6 per group). Overexpression of CDX2 in T1D mice cannot improve their hyperglycemia (**A**) but can reduce their 24h total urine microalbumin (**B**). (**C**, **D**) Positive staining density of Sirius red was analyzed in each group from 6 random fields (200×). Sirius red staining (**C**) and quantitative analysis (**D**) in each group.CDX2 can improve renal fibrosis induced by DKD. All data are mean±SD from three independent experiments. *n*=6; **P*<0.05 versus NC group; #*P*<0.05 versus DM group.
