# Supplementary material for: The role of CDX2 in renal tubular lesions during diabetic kidney disease
Source: Aging (Albany NY). 2021 Feb 17;13(5):6782–803. doi: 10.18632/aging.202537 (PMC7993706; doi:10.18632/aging.202537)
Supplement: Supplementary Table 1 [file aging-13-202537-s002.pdf]

SUPPLEMENTARY TABLE

Supplementary Table 1. Pathological diagnosis information of human kidney samples.

Patient ID	Gender	Age	Diagnosis
K180388	male	53	Tuberous sclerotic diabetic nephropathy(Tervaert classification III)
K180430	male	53	Tuberous sclerotic diabetic nephropathy(Tervaert classification III)
K180692	male	43	Tuberous sclerotic diabetic nephropathy(Tervaert classification III)
K1900006	male	35	Tuberous sclerotic diabetic nephropathy(Tervaert classification III)
K1900048	male	50	Tuberous sclerotic diabetic nephropathy(Tervaert classification III)Subacute tubular-interstitial injury
K1900025	female	59	Tuberous sclerotic diabetic nephropathy(Tervaert classification III)
K1804010	male	45	Splenic rupture and bleeding, left kidney rupture and bleeding (renal trauma)
K1835669	male	24	Left kidney rupture and bleeding (renal trauma)
K1912839	male	38	Right kidney rupture and bleeding (renal trauma)
K1924782	male	30	Left kidney rupture and bleeding (renal trauma)
K2001748	male	26	Left kidney rupture and bleeding (renal trauma)
K2008184	male	13	Right kidney rupture and bleeding (renal trauma)
